# Effect of Pharmacological Modulation of the Endocannabinoid System on Opiate Withdrawal: A Review of the Preclinical Animal Literature

**DOI:** 10.3389/fphar.2016.00187

**Published:** 2016-06-28

**Authors:** Kiri L. Wills, Linda A. Parker

**Affiliations:** Department of Psychology and Collaborative Neuroscience Program, University of Guelph, GuelphON, Canada

**Keywords:** cannabinoid, opioid, withdrawal, animal, amygdala, nucleus accumbens, locus coeruleus, periaqueductal gray

## Abstract

Over the years, animal studies have revealed a role for the endocannabinoid system in the regulation of multiple aspects of opiate addiction. The current review provides an overview of this literature in regards to opiate withdrawal. The opiate withdrawal syndrome, hypothesized to act as a negative reinforcer in mediating continued drug use, can be characterized by the emergence of spontaneous or precipitated aversive somatic and affective states following the termination of drug use. The behaviors measured to quantify somatic opiate withdrawal and the paradigms employed to assess affective opiate withdrawal (e.g., conditioned place aversion) in both acutely and chronically dependent animals are discussed in relation to the ability of the endocannabinoid system to modulate these behaviors. Additionally, the brain regions mediating somatic and affective opiate withdrawal are elucidated with respect to their modulation by the endocannabinoid system. Ultimately, a review of these findings reveals dissociations between the brain regions mediating somatic and affective opiate withdrawal, and the ability of cannabinoid type 1 (CB_1_) receptor agonism/antagonism to interfere with opiate withdrawal within different brain sub regions.

## Introduction

Opiate addiction is a chronic distressing brain disorder for which there are limited successful treatments that do not rely on the administration of synthetic opiate analogs (Stotts et al., 2009). One potential non-opioidergic therapeutic target is the endocannabinoid system. A review of the preclinical animal literature suggests that the endocannabinoid system is necessary for the development of opiate dependence and could prove to be useful in the treatment of opiate withdrawal.

## Cannabinoids On Opiate Withdrawal

In animals, as in humans, opioid dependence is produced through chronic opiate exposure, or even, following the single administration of a high dose of an opiate, a state termed acute opiate dependence (Heishman et al., 1989; June et al., 1995). Once dependent, withdrawal can be induced either spontaneously through drug abstinence, or precipitated through the administration of an opiate antagonist such as naloxone. In each instance, dependent animals will display numerous somatic and behavioral symptoms that are characteristic of the withdrawal state experienced by human opiate addicts (Jaffe, 1990). The ability of different pharmacological manipulations to alter the severity or presence of these symptoms can be used as a measure of their potential in the treatment of opiate detoxification.

### Somatic Withdrawal

In animals, the intensity of somatic withdrawal is quantified by scoring the presence or severity of several physical signs for 10–30 min immediately following precipitated withdrawal, or every 6–9 h for several days following spontaneous withdrawal (Maldonado et al., 1996). To facilitate the quantification of the withdrawal syndrome, Gellert and Holtzman (1978) developed a weighted scale consisting of graded symptoms including percentage of weight loss, number of escape jumps, number of wet dog shakes, number of abdominal constrictions, and checked signs including diarrhea, facial fasciculations/teeth chattering, swallowing, salivation, chromodacryorrhea, ptosis, abnormal posture, erection/ejaculation/genital grooming, and irritability. In assessing the ability of pharmacological cannabinoid manipulations to alleviate the intensity of somatic withdrawal, most studies use a variation of this scale to determine whether individual signs or a global rating of withdrawal has significantly decreased.

A review of the literature reveals that activation of the cannabinoid system acutely prior to withdrawal, or chronic inhibition of the system during the development of dependence, is most effective in reducing withdrawal severity (see **Table 1** for specific agonists and antagonists and original references). In the majority of cases, acute treatment with a cannabinoid agonist (in particular agonists of the CB_1_ receptor) immediately prior to the induction of withdrawal in highly dependent animals (whether precipitated or spontaneous) is able to attenuate several aspects of the syndrome. Notably, a reduction in the incidence of escape jumps, paw tremors, weight loss, and diarrhea were most commonly reported in rodents (see **Table 1** for original references). Exceptions to the reductions in withdrawal occurred only when the agonist (Δ^9^THC) was administered chronically during the development of morphine dependence (Cichewicz and Welch, 2003; Gerak and France, 2016), when rats were made acutely dependent on morphine prior to withdrawal (Hine et al., 1975b), or when the cannabinoid tested, cannabidiol, was not a CB_1_ receptor agonist (Hine et al., 1975d,e; Chesher and Jackson, 1985).

**Table 1:** Effect of different cannabinoid compounds on spontaneous or precipitated somatic and affective opioid withdrawal.

		Cannabinoid compound	Dosing	Effect on withdrawal	Animal	Reference
Somatic	Precipitated	**Agonists**				
		AEA	Acute	Reduced	Mice	Vela et al., 1995
		2-AG	Acute	Reduced	Mice	Yamaguchi et al., 2001
		Δ^9^ THC	Acute	Reduced	Mice	Gamage et al., 2015
			Acute	Reduced	Mice	Ramesh et al., 2011
			Acute/co-chronic	Reduced/enhanced	Mice	Cichewicz and Welch, 2003
			Acute	Reduced	Mice	Lichtman et al., 2001
			Pre-chronic	Reduced	Mice	Valverde et al., 2001
			Acute	Reduced	Mice	Vela et al., 1995
			Acute	Reduced	Rats	Chesher and Jackson, 1985^†^
			Acute	Reduced	Rats	Zaluzny et al., 1979^†^
			Acute	Reduced	Mice	Bhargava, 1976a,b
			Acute/co-chronic	Reduced	Guinea Pigs/rats	Frederickson et al., 1976
			Acute	Reduced/enhanced	Rats	Hine et al., 1975a,b,c,d,e
			*Acute*	*No effect*	*Rats*	*Hine et al., 1975b*
		Δ^8^ THC	Acute	Reduced	Mice	Yamaguchi et al., 2001
			Acute	Reduced	Mice	Bhargava, 1976a
		11 hydroxy – Δ^8^ THC	Acute	Reduced	Mice	Bhargava, 1976a
		CBD (non-CB_1_)	Acute	No effect	Rats	Chesher and Jackson, 1985^†^
			Acute	Reduced	Mice	Bhargava, 1976a
			Acute	No effect	Rats	Hine et al., 1975d,e
		CBN	Acute	Reduced	Rats	Chesher and Jackson, 1985^†^
			Acute	Reduced	Mice	Bhargava, 1976a
			Acute	No effect	Rats	Hine et al., 1975d
		HU-210	Acute	Reduced	Mice	Yamaguchi et al., 2001
		O-1602 (GPR55 agonist)	Co-chronic	Reduced	Mice	Alavi et al., 2016
		**AEA transport inhibitor**				
		AM404	Acute	No effect	Mice	Del Arco et al., 2002
		**FAAH inhibitors**				
		PF-3845	Acute	No effect	Mice	Gamage et al., 2015
			Acute	Reduced	Mice	Ramesh et al., 2011
		URB-597	Acute	Reduced	Rats	Shahidi and Hasanein, 2011
		**FAAH KO**		Reduced	Mice	Ramesh et al., 2011
		**MAGL inhibitor**				Gamage et al., 2015
		JZL-184	Acute	Reduced	Mice	Ramesh et al., 2011
		**Dual FAAH/MAGL**				
		**Inhibitors**				
		SA-57	Acute	Reduced	Mice	Gamage et al., 2015
		JZL-184 + PF-3845	Acute	Reduced	Mice	Ramesh et al., 2013
		**CB_1_ KO**		Reduced	Mice	Maccarrone et al., 2002
				Reduced	Mice	Lichtman et al., 2001
				Reduced	Mice	Ledent et al., 1999
		**CB_1_ antagonists/inverse agonists**				
		Rimonabant	Acute/co-chronic	Reduced aspects/reduced	Rats	Trang et al., 2006
			Acute/co-chronic	No effect/reduced	Mice	Mas-Nieto et al., 2001
			Co-chronic	Reduced/enhanced	Rats	Rubino et al., 2000
			Acute	No effect	Rats	Navarro et al., 1998
		AM251	Acute/co-chronic	No effect/reduced	Rats	Trang et al., 2006
	Spontaneous	**Agonists**				
		Δ^9^ THC	Co-chronic	No effect	Monkeys	Gerak and France, 2016
			Acute	Reduced	Dogs	Gilbert, 1981
		Nabilone	Acute	Reduced	Dogs	Gilbert, 1981
		Nantradol	Acute	Reduced	Dogs	Gilbert, 1981
			Acute	Reduced	Monkeys	Young et al., 1981
		Levonantradol	Acute	Reduced	Monkeys	Young et al., 1981
		**AEA transport inhibitor**				
		AM404	Acute	Reduced	Mice	Del Arco et al., 2002
		**MAGL inhibitor**				
		JZL-184	Acute	Reduced	Mice	Ramesh et al., 2011
		**Dual FAAH/MAGL inhibitors**				
		SA-57	Acute	Reduced	Mice	Ramesh et al., 2013
		JZL-184 + PF-3845	Acute	Reduced	Mice	Ramesh et al., 2013
		**CB_1_ antagonist/inverse agonist**				
		Rimonabant	Acute	Failed to precipitate	Mice	Lichtman et al., 2001
			Acute	Precipitated	Rats	Navarro et al., 1998

Affective	CPA paradigm	**Agonists**				


		Δ^9^ THC	Acute	No effect	Mice	Gamage et al., 2015
		**FAAH inhibitors**				
		PF-3845	Acute	No effect	Mice	Gamage et al., 2015
			*Acute*	*No effect*	*Rats*	*Wills et al., 2014*
		URB-597	*Acute*	*No effect*	*Rats*	*Wills et al., 2014*
		**MAGL inhibitors**				
		JZL-184	Acute	No effect	Mice	Gamage et al., 2015
		MJN110	*Acute*	*Reduced*	*Rats*	*Wills et al., 2014, 2016*
		**Dual FAAH/MAGL inhibitor**				
		SA-57	Acute	No effect	Mice	Gamage et al., 2015
		**CB_1_ KO**		No effect	Mice	Martin et al., 2000
				Reduced	Mice	Ledent et al., 1999
		**CB_1_ antagonists/inverse agonists**				
		Rimonabant	Acute	Precipitated	Rats	Navarro et al., 2001^‡^
		AM251	*Acute*	*Reduced*	*Rats*	*Wills et al., 2014, 2016*
		**CB_1_ neutral antagonist**				
		AM4113	*Acute*	*Reduced*	*Rats*	*Wills et al., 2014*
		AM6527	*Acute*	*Reduced*	*Rats*	*Wills et al., 2014*
	Operant responding for food	**CB_1_ antagonist/inverse agonist**				


		Rimonabant	Acute	Precipitated	Rats	Navarro et al., 2001^‡^


*Dosing reflects whether compounds were given acutely prior to withdrawal, pre-chronic (chronically prior to morphine administration), or co-chronic (concurrently with morphine during the development of dependence). *Italicized rows* indicate experiments tested on acutely dependent animals. ^†^Quasi morphine withdrawal, ^‡^ spontaneous withdrawal.*

Somewhat contradictory, CB_1_ receptor knock-out (KO) mice show reduced somatic opioid withdrawal and cannabinoid antagonists (rimonabant and AM251) interfere with somatic opioid withdrawal (see **Table 1**). However, contrary to the cannabinoid agonists, antagonists were most effective in reducing withdrawal when delivered chronically (not acutely) during the development of opiate dependence. Indeed, when given chronically prior to precipitating withdrawal with naloxone, rimonabant and AM251 reliably reduced withdrawal severity (Rubino et al., 2000; Mas-Nieto et al., 2001; Trang et al., 2006). In contrast, when delivered acutely, antagonists were either without effect (Navarro et al., 1998; Mas-Nieto et al., 2001; Trang et al., 2006), or their ability to attenuate withdrawal was markedly reduced (Trang et al., 2006). In a case of spontaneous withdrawal (Navarro et al., 1998), acute rimonabant treatment was even found to precipitate withdrawal in morphine-dependent rats, however, this finding was not replicated when tested in mice (Lichtman et al., 2001). Ultimately, these findings, along with the results from the agonist studies, suggest that the endocannabinoid system plays an important role in the development of somatic opioid dependence and activation of the system during withdrawal, or chronic blockade during opioid dependence, can mitigate some of its adverse effects.

Unfortunately, CB_1_ receptor (CB_1_R) agonists and inverse agonists/antagonists are known to produce undesirable side effects (e.g., psychoactivity, depression) which limit their therapeutic potential (Moreira et al., 2009). Therefore, it is fortunate that non-psychoactive treatments (including Fatty Acid Amide Hydrolase [FAAH] and monoacylglycerol lipase [MAGL] inhibitors) which act to enhance endogenous cannabinoid tone have also been effective in alleviating somatic withdrawal (see **Table 1** for specific agents and original references). While inhibition of the MAGL enzyme (which elevates endogenous 2-arachidonoyl glycerol [2-AG]) produced the most robust effects (Ramesh et al., 2011; Gamage et al., 2015), inhibitors or KO mice of the catabolic FAAH enzyme (which elevates endogenous anandamide [AEA]) reduced a subset of withdrawal symptoms in most cases (Ramesh et al., 2011; Shahidi and Hasanein, 2011), whereas the AEA transport inhibitor, AM404, was without effect (Del Arco et al., 2002). However, unlike FAAH inhibitors, MAGL inhibitors have been found to produce cannabimimetic side effects (e.g., hypomotility, hyperreflexia; Long et al., 2009) and can lead to the development of dependence and tolerance with repeated administration (Schlosburg et al., 2010). In light of this, Ramesh et al. (2013) and Gamage et al. (2015) tested the combinations of low doses of FAAH and MAGL inhibitors, or dual FAAH/MAGL inhibitors, for their effectiveness in reducing withdrawal maximally without additional side effects. Indeed, this combination of catabolic enzyme inhibitors proved to be highly effective in reducing withdrawal (including jumping, paw flutters, head shakes, diarrhea, and weight loss) but was absent of adverse effects. Consequently, when considering pharmacological interventions that may aid in the treatment of somatic aspects of opiate withdrawal, dual FAAH/MAGL inhibition (at low doses) is most promising.

### Affective Withdrawal

In animals, affective opioid withdrawal can be measured using a number of motivational paradigms including the conditioned place aversion (CPA), intracranial self-stimulation, and operant responding for food (Maldonado et al., 1996). In evaluating the role of the endocannabinoid system in affective opioid withdrawal, the CPA paradigm has been most commonly employed. This paradigm typically involves pairing naloxone-precipitated morphine withdrawal (in acutely or chronically dependent animals) with a specific environmental context, such that, upon re-exposure to this context in a drug-free state, animals will preferentially avoid the withdrawal paired context versus a context that was previously paired with a placebo saline injection (Sanchis-Segura and Spanagel, 2006). This avoidance of the withdrawal context is used as a measure of the intensity of the aversive affect experienced during opioid withdrawal, and can be used to assess the potential of pharmacological treatments to reduce affective withdrawal. Indeed, pharmacological treatments that are currently used in the treatment of opioid withdrawal (e.g., buprenorphine) are effective in reducing the establishment of avoidance behavior in this paradigm (Stinus et al., 2005). Also employed is the operant responding for food paradigm (Navarro et al., 2001). In this paradigm, a pharmacological treatment is deemed effective in reducing affective withdrawal if it is able to suppress a withdrawal-induced reduction in operant responding for food (Maldonado et al., 1996).

A review of the literature reveals a more complicated role for the endocannabinoid system in its ability to alleviate affective opioid withdrawal than was observed for somatic withdrawal (see **Table 1**); granted the studies conducted thus far are fairly limited. Indeed, as with somatic withdrawal, both activation and inhibition of the cannabinoid system has been found effective in reducing affective opioid withdrawal, but with greater inconsistencies. Contrary to somatic withdrawal, the cannabinoid agonist, Δ^9^THC, and the FAAH enzyme inhibitors, PF3845 and URB-597, tested were unable to modify the establishment of a naloxone-precipitated CPA (Wills et al., 2014; Gamage et al., 2015). However, while activation of the cannabinoid system via exogenous cannabinoid administration or endogenous elevation of AEA proved to have little efficacy, inhibition of MAGL activity and concomitant elevation of 2-AG showed mixed (Wills et al., 2014, 2016; Gamage et al., 2015) but more promising results. Given that these discrepant findings were obtained by different laboratories using different procedures, compounds (JZL-184 vs. MJN110), and species (mice vs. rats), more research into the potential of MAGL inhibition in reducing affective opioid withdrawal will be required in order to elucidate its role. Furthermore, while the only dual FAAH/MAGL inhibitor (SA-57) investigated was unable to modify a naloxone-precipitated CPA (Gamage et al., 2015), additional research into the ability of such compounds to reduce affective withdrawal should continue owing to their effectiveness in reducing somatic withdrawal with minor adverse effects (noted in previous section).

Although the deleterious reputation of cannabinoid antagonists/inverse agonists may limit their therapeutic potential (Bergman et al., 2008), blockade of the endocannabinoid system may be effective for reducing affective opioid withdrawal. Indeed, acute CB_1_R antagonism (with both AM251, and the neutral CB_1_ antagonists, AM4113, and AM6527) was effective in preventing the establishment of a naloxone-precipitated CPA (Wills et al., 2014, 2016). This is in contrast to the ability of acute CB_1_ antagonism to reliably reduce somatic withdrawal, but could be attributed to the fact that the CPA paradigm may provide a more sensitive measure of opioid withdrawal (Azar et al., 2003). Of special consideration is that both antagonists (which have been found to possess inverse agonist activity) and neutral antagonists (void of intrinsic activity; Sink et al., 2008) were effective in preventing the establishment of the CPA. This is particularly important since these ‘neutral’ antagonists have also been found to lack the adverse qualities attributed to typical cannabinoid antagonists (Bergman et al., 2008). While the only evaluations of chronic inhibition of the endocannabinoid system (CB_1_ KO mice) yielded mixed results (Ledent et al., 1999; Martin et al., 2000), chronic antagonism of the endocannabinoid system during the development of dependence using ‘neutral’ antagonists should be investigated given that acute treatments have been reported to precipitate both spontaneous affective and somatic withdrawal (Navarro et al., 1998, 2001).

## Neurobiological Correlates and Potential Mechanisms of Interaction

Although the exact mechanisms through which the endocan nabinoid system is able to modulate opioid withdrawal remain to be elucidated, several theories have been proposed. Briefly, it has been hypothesized that cannabinoid and opioids interact through (1) reciprocal endogenous neurotransmitter/peptide release, (2) common signal-transduction pathways, and (3) receptor heterodimerization (see Parolaro et al., 2010; Scavone et al., 2013, for a review). An overview of these mechanisms in the brain regions most attributed to somatic and affective withdrawal is discussed.

### Somatic Withdrawal

The neuroanatomical substrates most sensitive to the appearance of somatic opioid withdrawal include the periaqueductal gray (PAG) and locus coeruleus (LC; see Maldonado et al., 1996, for a review). Indeed, microinjection of methylnaloxonium, a hydrophilic opiate antagonist, into these regions was most sensitive in eliciting the opiate withdrawal syndrome, with active symptoms such as jumping being particularly prominent (Maldonado et al., 1992). In revisiting the withdrawal symptoms most commonly attenuated by cannabinoid modulation (e.g., jumping and paw tremors), it becomes apparent that these neuroanatomical substrates may represent regions through which cannabinoids interact with the opioid system to attenuate somatic withdrawal.

Much evidence indicates the PAG as a locus for cannabinoid–opioid interactions. Indeed, anatomical immunolabeling has co-localized the CB_1_R and μ-opioid receptor (MOR) within this region. Furthermore, 8% of immunoreactive MOR PAG neurons received immunoreactive CB_1_R appositions, indicating a role for presynaptic cannabinoid modulation (Wilson-Poe et al., 2012). In addition, sub-chronic CB_1_R agonist treatment with THC or AM356 was reported to increase proenkephalin mRNA levels in the PAG (Manzanares et al., 1998). A reciprocal effect following repeated morphine treatment is also suggested given the ability of the CB_1_R antagonist AM251 to increase the frequency of spontaneous miniature inhibitory postsynaptic currents (IPSCs) in morphine, but not saline treated animals, suggesting an elevation of endocannabinoid tone (Wilson-Poe et al., 2014). Chronic (up to 7 days) CB_1_R agonist treatment has also been reported to up-regulate MOR density (Viganò et al., 2005), with tolerance developing with prolonged (14 day) treatment (Corchero et al., 2004). Finally, chronic THC pre-opiate exposure or acute rimonabant treatment has been found to induce or attenuate Fos immunoreactivity in the PAG from acute heroin or morphine administration, respectively (Singh et al., 2004, 2005).

While the investigation of cannabinoid–opioid interaction in the LC is less extensive than in the PAG, a recent anatomical study confirmed the co-existence of MOR and CB_1_R immunoreactivity in somatodendritic compartments of catecholaminergic neurons. Additionally, as in the PAG, immunoreactive CB_1_R axon terminals formed synaptic contacts with MOR dendrites (Scavone et al., 2010). Ultimately, these findings suggest a role for cannabinoid–opioid interactions in the PAG and LC via common signal transduction mechanisms, or potential receptor heterodimerization given their close proximity and reports of this occurrence (Schoffelmeer et al., 2006). Additionally, as discussed above, interactions in endogenous neurotransmitter release are also evident in the PAG. These mechanisms of interaction correspond to the general ability of CB_1_R agonism or chronic CB_1_R antagonism to reduce symptoms of somatic opioid withdrawal.

### Affective Withdrawal

The brain structures most sensitive to the motivating or affective component of opioid withdrawal include the nucleus accumbens (NAc) and the amygdala (see Maldonado et al., 1996, for a review). Indeed, microinjection of hydrophilic opiate antagonists into these regions was most sensitive in producing the establishment of a CPA (Stinus et al., 1990), and suppressing operant responding in morphine dependent rats (Koob et al., 1989). An evaluation of cannabinoid–opioid interactions within these regions also reveals these sites to be crucial in the ability of the cannabinoid system to modulate affective opioid withdrawal.

There is substantial research supporting cannabinoid–opioid interactions within the NAc. As in the PAG and LC, immunolabeling has revealed evidence for CB_1_R and MOR localization on the same neurons in the NAc shell and core, as well as expression on synaptically linked neurons (Pickel et al., 2004). Furthermore, CB_1_R and MOR allosterically interact in this region causing non-additive and synergistic effects on glutamate and GABA release, respectively. These effects were reversed by their respective antagonists, but antagonist co-administration blocked these antagonistic effects, suggesting the potential for G-protein coupled heterodimeric receptor complexes (Schoffelmeer et al., 2006). Consistent with these findings, reports also suggest CB_1_R and MOR synergy on cAMP/PKA signaling that is mediated through βγ dimers (Yao et al., 2006). Chronic opioid and cannabinoid administration has also been found to alter cannabinoid and opioid stimulated G-protein receptor coupling, respectively; though while cannabinoids enhance MOR binding (Viganò et al., 2005), opioids have produced more mixed effects on CB_1_R binding (Viganò et al., 2003, 2005; Fattore et al., 2007). Reports of changes in receptor densities have also been noted, with chronic opioid administration increasing CB_1_R density (Gonzalez et al., 2002), mRNA and protein levels (Ren et al., 2009), and cannabinoid agonist treatment increasing MOR density (Corchero et al., 2004; Fattore et al., 2007; Molaei et al., 2016). However, while cannabinoid agonists seem to consistently increase MOR density, the effects of CB_1_R gene deletion have been less consistent in reducing expressivity (Urigüen et al., 2005; Lane et al., 2010). Cannabinoid–opioid interactions on neuronal activation and dopamine release have also been reported in the NAc. Indeed, cannabinoid agonists increase while antagonists decrease Fos expression induced by acute morphine (Singh et al., 2004, 2005), and CB_1_R KO mice report decreased morphine-induced dopamine release (Mascia et al., 1999). Finally, cross-talk on endogenous neurotransmitter and peptide release has also been described, with increased AEA and decreased 2-AG content following chronic opioid administration, and increased Met-enkephalin immunoreactivity (Valverde et al., 2001), proenkephalin mRNA (Manzanares et al., 1998), and β-endorphin levels (Solinas et al., 2004) following acute or chronic cannabinoid (THC or CP-55,940) administration. These findings are consistent with the ability of cannabinoid agonists to decrease affective opioid withdrawal and provide evidence for interactions within the NAc at the neurotransmitter, receptor and signal transduction level.

A second region of interest attributed to mediating affective opioid withdrawal is the amygdala. Although there is less research investigating cannabinoid–opioid interactions within this region in comparison to the NAc, effects on receptor density and functionality have been noted. Specifically, acute and repeated cannabinoid agonist (THC or WIN 55,212-2) administration produced increases in MOR density and G-protein receptor coupling in the amygdala (Corchero et al., 2004; Fattore et al., 2007). Similar findings were also reported on the effects of chronic opioid administration on CB_1_R density and receptor coupling when considering the amygdala as a whole (Fattore et al., 2007), however, decreases in CB_1_R density have been described when analyzing the basolateral amygdala (BLA) individually (Gonzalez et al., 2002). In addition to receptor interactions, alterations in Fos immunoreactivity (Fos IR; a marker of neuronal activation) have also been noted. Indeed, acute treatment of the cannabinoid antagonist, rimonabant, attenuated acute morphine-induced Fos immunoreactivity in the amygdala as a whole (Singh et al., 2004), while investigations of the central nucleus of the amygdala (CeA) specifically revealed decreases in acute heroin-induced Fos IR following chronic THC pre-treatment (Singh et al., 2005), and additive effects on naloxone-induced Fos IR following acute THC exposure (Allen et al., 2003). Although the above studies do not make any direct comparisons between the BLA and CeA on cannabinoid–opioid interactions, a recent experiment suggests a functional double dissociation between these regions in the ability of the cannabinoid system to modulate affective opioid withdrawal. Indeed, cannabinoid agonism (via inhibition of MAGL activity with MJN110) within the BLA, while cannabinoid antagonism (AM251) within the CeA, is able to interfere with the establishment of a naloxone-precipitated CPA in acutely morphine dependent rats (Wills et al., 2016). Though the mechanisms mediating the dissociation between these regions were not investigated, this finding suggests potential differences in cannabinoid–opioid modulation within different sub-regions of the amygdala.

## Conclusion

A review of the literature reveals that acute pharmacological activation or chronic inhibition of the cannabinoid system may interfere with opioid withdrawal. In particular, dual FAAH/MAGL enzyme inhibitors or neutral CB_1_R antagonists are most promising, though further investigations are necessary. In the central nervous system, cannabinoid–opioid interactions are abundant and present within neuroanatomical regions important in mediating opioid withdrawal including the amygdala, the NAc, the PAG, and the LC. However, while interactions have been described, more research on the mechanisms through which endocannabinoid modulation is able to attenuate opioid withdrawal will be required in order to gain a more cohesive understanding of its therapeutic potential.

## Author Contributions

KW wrote the article; LP edited the article.

## Conflict of Interest Statement

The authors declare that the research was conducted in the absence of any commercial or financial relationships that could be construed as a potential conflict of interest.

## References

[B1] Alavi M. S. Hosseinzadeh H. Shamsizadeh A. Roohbakhsh A. (2016). The effect of O-1602, an atypical cannabinoid, on morphine-induced conditioned place preference and physical dependence. *Pharmacol. Rep.* 68 592–597. 10.1016/j.pharep.2015.12.009 26971034

[B2] Allen K. V. McGregor I. S. Hunt G. E. Singh M. E. Mallet P. E. (2003). Regional differences in naloxone modulation of Δ9-THC induced Fos expression in rat brain. *Neuropharmacology* 44 264–272. 10.1016/S0028-3908(02)00364-7 12623225

[B3] Azar M. R. Jones B. C. Schulteis G. (2003). Conditioned place aversion is a highly sensitive index of acute opioid dependence and withdrawal. *Psychopharmacology* 170 42–50. 10.1007/s00213-003-1514-y 12783156

[B4] Bergman J. Delatte M. S. Paronis C. A. Vemuri K. Thakur G. A. Makriyannis A. (2008). Some effects of CB1 antagonists with inverse agonist and neutral biochemical properties. *Physiol. Behav.* 93 666–670. 10.1016/j.physbeh.2007.11.007 18076956 PMC2441972

[B5] Bhargava H. N. (1976a). Effect of some cannabinoids on naloxone-precipitated abstinence in morphine-dependent mice. *Psychopharmacology* 49 267–270. 10.1007/BF00426828 826944

[B6] Bhargava H. N. (1976b). Inhibition of naloxone-induced withdrawal in morphine dependent mice by l-trans-Δ9-tetrahydrocannabinol. *Eur. J. Pharmacol.* 36 259–262. 10.1016/0014-2999(76)90283-1 944134

[B7] Chesher G. B. Jackson D. M. (1985). The quasi-morphine withdrawal syndrome: effect of cannabinol, cannabidiol and tetrahydrocannabinol. *Pharmacol. Biochem. Behav.* 23 13–15. 10.1016/0091-3057(85)90122-4 2994117

[B8] Cichewicz D. L. Welch S. P. (2003). Modulation of oral morphine antinociceptive tolerance and naloxone-precipitated withdrawal signs by oral Delta 9-tetrahydrocannabinol. *J. Pharmacol. Exp. Ther.* 305 812–817. 10.1124/jpet.102.046870 12606610

[B9] Corchero J. Oliva J. M. García-Lecumberri C. Martin S. Ambrosio E. Manzanares J. (2004). Repeated administration with Δ9-tetrahydrocannabinol regulates μ-opioid receptor density in the rat brain. *J. Psychopharmacol.* 18 54–58. 10.1177/0269881104040237 15107185

[B10] Del Arco I. Navarro M. Bilbao A. Ferrer B. Piomelli D. Rodríguez De Fonseca F. (2002). Attenuation of spontaneous opiate withdrawal in mice by the anandamide transport inhibitor AM404. *Eur. J. Pharmacol.* 454 103–104. 10.1016/S0014-2999(02)02483-4 12409011

[B11] Fattore L. Viganò D. Fadda P. Rubino T. Fratta W. Parolaro D. (2007). Bidirectional regulation of mu-opioid and CB1-cannabinoid receptor in rats self-administering heroin or WIN 55,212-2. *Eur. J. Neurosci.* 25 2191–2200. 10.1111/j.1460-9568.2007.05470.x 17419755

[B12] Frederickson R. C. Hewes C. R. Aiken J. W. (1976). Correlation between the in vivo and an in vitro expression of opiate withdrawal precipitated by naloxone: their antagonism by l-(-)-delta9-tetrahydrocannabinol. *J. Pharmacol. Exp. Ther.* 199 375–384. 988178

[B13] Gamage T. F. Ignatowska-Jankowska B. M. Muldoon P. P. Cravatt B. F. Damaj M. I. Lichtman A. H. (2015). Differential effects of endocannabinoid catabolic inhibitors on morphine withdrawal in mice. *Drug Alcohol Depend.* 146 7–16. 10.1016/j.drugalcdep.2014.11.015 25479915 PMC4295928

[B14] Gellert V. F. Holtzman S. G. (1978). Development and maintenance of morphine tolerance and dependence in the rat by scheduled access to morphine drinking solutions. *J. Pharmacol. Exp. Ther.* 205 536–546. 566320

[B15] Gerak L. R. France C. P. (2016). Combined treatment with morphine and 9-tetrahydrocannabinol in rhesus monkeys: antinociceptive tolerance and withdrawal. *J. Pharmacol. Exp. Ther.* 357 357–366. 10.1124/jpet.115.231381 26937020 PMC4851324

[B16] Gilbert P. E. (1981). A comparison of THC, nantradol, nabilone, and morphine in the chronic spinal dog. *J. Clin. Pharmacol.* 21(8–9 Suppl.), 311S–319S. 10.1002/j.1552-4604.1981.tb02609.x 6271835

[B17] Gonzalez S. Fernandez J. Parolaro D. (2002). Chronic exposure to morphine, cocaine or ethanol in rats produced different effects in brain cannabinoid CB 1 receptor binding and mRNA levels. *Drug Alcohol Depend.* 66 77–84. 10.1016/S0376-8716(01)00186-7 11850139

[B18] Heishman S. J. Stitzer M. L. Bigelow G. E. Liebson I. A. (1989). Acute opioid physical dependence in postaddict humans: naloxone dose effects after brief morphine exposure. *J. Pharmacol. Exp. Ther.* 248 127–134. 2913267

[B19] Hine B. Friedman E. Torrelio M. Gershon S. (1975a). Morphine-dependent rats: blockade of precipitated abstinence by tetrahydrocannabinol. *Science* 187 443–445. 10.1126/science.1167428 1167428

[B20] Hine B. Friedman E. Torrelio M. Gershon S. (1975b). Tetrahydrocannabinol-attenuated abstinence and induced rotation in morphine-dependent rats: possible involvement of dopamine. *Neuropharmacology* 14 607–610. 10.1016/0028-3908(75)90128-8 1237098

[B21] Hine B. Torrelio M. Gershon S. (1975c). Attenuation of precipitated abstinence in methadone-dependent rats by delta 9-THC. *Psychopharmacol. Commun.* 1 275–283. 1241452

[B22] Hine B. Torrelio M. Gershon S. (1975d). Differential effect of cannabinol and cannabidiol on THC-induced responses during abstinence in morphine-dependent rats. *Res. Commun. Chem. Pathol. Pharmacol.* 12 185–188. 1237925

[B23] Hine B. Torrelio M. Gershon S. (1975e). Interactions between cannabidiol and Δ9-THC during abstinence in morphine-dependent rats. *Life Sci.* 17 851–857. 10.1016/0024-3205(75)90435-X 1238886

[B24] Jaffe J. H. (1990). “Drug addiction and drug abuse,” in *Goodman and Gilman’s the Pharmacological Basis of Therapeutics*, ed. Brunton L. L. (New York, NY: McGraw-Hill Education), 522–573.

[B25] June H. L. Stitzer M. L. Cone E. (1995). Acute physical dependence: time course and relation to human plasma morphine concentrations. *Clin. Pharmacol. Ther.* 57 270–280. 10.1016/0009-9236(95)90152-3 7697945

[B26] Koob G. F. Wall T. L. Bloom F. E. (1989). Nucleus accumbens as a substrate for the aversive stimulus effects of opiate withdrawal. *Psychopharmacology* 98 530–534. 10.1007/BF00441954 2505294

[B27] Lane D. A. Chan J. Lupica C. R. Pickel V. M. (2010). Cannabinoid-1 receptor gene deletion has a compartment-specific affect on the dendritic and axonal availability of μ-opioid receptors and on dopamine axons in the mouse nucleus accumbens. *Synapse* 64 886–897. 10.1002/syn.20807 20939059 PMC2954666

[B28] Ledent C. Valverde O. Cossu G. Petitet F. Aubert J. F. Beslot F. (1999). Unresponsiveness to cannabinoids and reduced addictive effects of opiates in CB1 receptor knockout mice. *Science* 283 401–404. 10.1126/science.283.5400.401 9888857

[B29] Lichtman A. H. Sheikh S. M. Loh H. H. Martin B. R. (2001). Opioid and cannabinoid modulation of precipitated withdrawal in delta(9)-tetrahydrocannabinol and morphine-dependent mice. *J. Pharmacol. Exp. Ther.* 298 1007–1014. 11504797

[B30] Long J. Z. Li W. Booker L. Burston J. J. Kinsey S. G. Schlosburg J. E. (2009). Selective blockade of 2-arachidonoylglycerol hydrolysis produces cannabinoid behavioral effects. *Nat. Chem. Biol.* 5 37–44. 10.1038/nchembio.129 19029917 PMC2605181

[B31] Maccarrone M. Valverde O. Barbaccia M. L. Castañé A. Maldonado R. Ledent C. (2002). Age-related changes of anandamide metabolism in CB1 cannabinoid receptor knockout mice: correlation with behaviour. *Eur. J. Neurosci.* 15 1178–1186. 10.1046/j.1460-9568.2002.01957.x 11982628

[B32] Maldonado R. Stinus L. Gold L. H. Koob G. F. (1992). Role of different brain structures in the expression of the physical morphine withdrawal syndrome. *J. Pharmacol. Exp. Ther.* 261 669–677. 1578378

[B33] Maldonado R. Stinus L. Koob G. F. (1996). *Neurobiological Mechanisms of Opiate Withdrawal.* Austin, TX: R.G. Landes Company.

[B34] Manzanares J. Corchero J. Romero J. Fernandez-Ruiz J. J. Ramos J. A. Fuentes J. A. (1998). Chronic administration of cannabinoids regulates proenkephalin mRNA levels in selected regions of the rat brain. *Mol. Brain Res.* 55 126–132. 10.1016/S0169-328X(97)00371-9 9645967

[B35] Martin M. Ledent C. Parmentier M. Maldonado R. Valverde O. (2000). Cocaine, but not morphine, induces conditioned place preference and sensitization to locomotor responses in CB1 knockout mice. *Euro. J. Neurosci.* 12 4038–4046. 10.1046/j.1460-9568.2000.00287.x 11069600

[B36] Mascia M. S. Obinu M. C. Ledent C. Parmentier M. Böhme G. A. Imperato A. (1999). Lack of morphine-induced dopamine release in the nucleus accumbens of cannabinoid CB1 receptor knockout mice. *Eur. J. Pharmacol.* 383 R1–R2. 10.1016/S0014-2999(99)00656-1 10594337

[B37] Mas-Nieto M. Pommier B. Tzavara E. T. Caneparo A. Le Fur G. Roques B. P. (2001). Reduction of opioid dependence by the CB (1) antagonist SR141716A in mice: evaluation of the interest in pharmacotherapy of opioid addiction. *Br. J. Pharmacol.* 132 1809–1816. 10.1038/sj.bjp.0703990 11309253 PMC1572728

[B38] Molaei M. Fatahi Z. Zaringhalam J. Haghparast A. (2016). CB1 cannabinoid agonist (WIN55,212-2) within the basolateral amygdala induced sensitization to morphine and increased the level of μ-Opioid receptor and c-fos in the nucleus accumbens. *J. Mol. Neurosci.* 58 446–455. 10.1007/s12031-016-0716-9 26803309

[B39] Moreira F. A. Grieb M. Lutz B. (2009). Central side-effects of therapies based on CB1 cannabinoid receptor agonists and antagonists: focus on anxiety and depression. *Best Pract. Res. Clin. Endocrinol. Metab.* 23 133–144. 10.1016/j.beem.2008.09.003 19285266

[B40] Navarro M. Carrera M. R. Fratta W. Valverde O. Cossu G. Fattore L. (2001). Functional interaction between opioid and cannabinoid receptors in drug self-administration. *J. Neurosci.* 21 5344–5350. 11438610 10.1523/JNEUROSCI.21-14-05344.2001 PMC6762870

[B41] Navarro M. Chowen C. A. J. Arco I. Villanúa M. A. Martin Y. Roberts A. J. (1998). CB 1 cannabinoid receptor antagonist-induced opiate withdrawal in morphine-dependent rats. *Neuropharmacology* 9 3397–3402. 10.1097/00001756-199810260-00012 9855288

[B42] Parolaro D. Rubino T. Vigano D. Massi P. Guidali C. Realini N. (2010). Cellular mechanisms underlying the interaction between cannabinoid and opioid system. *Curr. Drug Targets* 11 393–405. 10.2174/138945010790980367 20017730

[B43] Pickel V. M. Chan J. Kash T. L. Rodriguez J. J. MacKie K. (2004). Compartment-specific localization of cannabinoid 1 (CB1) and mu-opioid receptors in rat nucleus accumbens. *Neuroscience* 127 101–112. 10.1016/j.neuroscience.2004.05.015 15219673

[B44] Ramesh D. Gamage T. F. Vanuytsel T. Owens R. A. Abdullah R. A. Niphakis M. J. (2013). Dual inhibition of endocannabinoid catabolic enzymes produces enhanced antiwithdrawal effects in morphine-dependent mice. *Neuropsychopharmacology* 38 1039–1049. 10.1038/npp.2012.269 23303065 PMC3629394

[B45] Ramesh D. Ross G. R. Schlosburg J. E. Owens R. A. Abdullah R. A. Kinsey S. G. (2011). Blockade of endocannabinoid hydrolytic enzymes attenuates precipitated opioid withdrawal symptoms in mice. *J. Pharmacol. Exp. Ther.* 339 173–185. 10.1124/jpet.111.181370 21719468 PMC3186294

[B46] Ren Y. Whittard J. Higuera-Matas A. Morris C. V. Hurd Y. L. (2009). Cannabidiol, a nonpsychotropic component of cannabis, inhibits cue-induced heroin seeking and normalizes discrete mesolimbic neuronal disturbances. *J. Neurosci.* 29 14764–14769. 10.1523/JNEUROSCI.4291-09.2009 19940171 PMC2829756

[B47] Rubino T. Massi P. Vigano D. Fuzio D. Parolaro D. (2000). Long-term treatment with SR141716A, the CB1 receptor antagonist, influences morphine withdrawal syndrom. *Life Sci.* 66 2213–2219. 10.1016/S0024-3205(00)00547-6 10834304

[B48] Sanchis-Segura C. Spanagel R. (2006). Behavioural assessment of drug reinforcement and addictive features in rodents: an overview. *Addict. Biol.* 11 2–38. 10.1111/j.1369-1600.2006.00012.x 16759333

[B49] Scavone J. L. Mackie K. Van Bockstaele E. J. (2010). Characterization of cannabinoid-1 receptors in the locus coeruleus: relationship with mu-opioid receptors. *Brain Res.* 1312 18–31. 10.1016/j.brainres.2009.11.023 19931229 PMC2835571

[B50] Scavone J. L. Sterling R. C. Van Bockstaele E. J. (2013). Cannabinoid and opioid interactions: implications for opiate dependence and withdrawal. *Neuroscience* 248 637–654. 10.1016/j.neuroscience.2013.04.034 23624062 PMC3742578

[B51] Schlosburg J. E. Blankman J. L. Long J. Z. Nomura D. K. Pan B. Kinsey S. G. (2010). Chronic monoacylglycerol lipase blockade causes functional antagonism of the endocannabinoid system. *Nat. Neurosci.* 13 1113–1119. 10.1038/nn.2616 20729846 PMC2928870

[B52] Schoffelmeer A. N. Hogenboom F. Wardeh G. De Vries T. J. (2006). Interactions between CB1 cannabinoid and mu opioid receptors mediating inhibition of neurotransmitter release in rat nucleus accumbens core. *Neuropharmacology* 51 773–781. 10.1016/j.neuropharm.2006.05.019 16806307

[B53] Shahidi S. Hasanein P. (2011). Behavioral effects of fatty acid amide hydrolase inhibition on morphine withdrawal symptoms. *Brain Res. Bull.* 86 118–122. 10.1016/j.brainresbull.2011.06.019 21763761

[B54] Singh M. E. McGregor I. S. Mallet P. E. (2005). Repeated exposure to Δ9-tetrahydrocannabinol alters heroin-induced locomotor sensitisation and Fos-immunoreactivity. *Neuropharmacology* 49 1189–1200. 10.1016/j.neuropharm.2005.07.008 16137723

[B55] Singh M. E. Verty A. N. A. Price I. McGregor I. S. Mallet P. E. (2004). Modulation of morphine-induced Fos-immunoreactivity by the cannabinoid receptor antagonist SR 141716. *Neuropharmacology* 47 1157–1169. 10.1016/j.neuropharm.2004.08.008 15567425

[B56] Sink K. S. McLaughlin P. J. Wood J. A. T. Brown C. Fan P. Vemuri V. K. (2008). The novel cannabinoid CB1 receptor neutral antagonist AM4113 suppresses food intake and food-reinforced behavior but does not induce signs of nausea in rats. *Neuropsychopharmacology* 33 946–955. 10.1038/sj.npp.1301476 17581535 PMC3711240

[B57] Solinas M. Zangen A. Thiriet N. Goldberg S. R. (2004). β-Endorphin elevations in the ventral tegmental area regulate the discriminative effects of Δ-9-tetrahydrocannabinol. *Eur. J. Neurosci.* 19 3183–3192. 10.1111/j.0953-816X.2004.03420.x 15217374

[B58] Stinus L. Cador M. Zorrilla E. P. Koob G. F. (2005). Buprenorphine and a CRF1 antagonist block the acquisition of opiate withdrawal-induced conditioned place aversion in rats. *Neuropsychopharmacology* 30 90–98. 10.1038/sj.npp.1300487 15138444

[B59] Stinus L. Le Moal M. Koob G. F. (1990). Nucleus accumbens and amygdala are possible substrates for the aversive stimulus effects of opiate withdrawal. *Neuroscience* 37 767–773. 10.1016/0306-4522(90)90106-E 2247222

[B60] Stotts A. L. Dodrill C. L. Kosten T. R. (2009). Opioid dependence treatment: options in pharmacotherapy. *Expert Opin. Pharmacother.* 10 1727–1740. 10.1517/14656560903037168 19538000 PMC2874458

[B61] Trang T. Ma W. Chabot J.-G. Quirion R. Jhamandas K. (2006). Spinal modulation of calcitonin gene-related peptide by endocannabinoids in the development of opioid physical dependence. *Pain* 126 256–271. 10.1016/j.pain.2006.07.008 16935424

[B62] Urigüen L. Berrendero F. Ledent C. Maldonado R. Manzanares J. (2005). Kappa- and delta-opioid receptor functional activities are increased in the caudate putamen of cannabinoid CB1 receptor knockout mice. *Eur. J. Neurosci.* 22 2106–2110. 10.1111/j.1460-9568.2005.04372.x 16262648

[B63] Valverde O. Noble F. Beslot F. Daugé V. Fournié-Zaluski M. C. Roques B. P. (2001). Δ9-tetrahydrocannabinol releases and facilitates the effects of endogenous enkephalins: reduction in morphine withdrawal syndrome without change in rewarding effect. *Eur. J. Neurosci.* 13 1816–1824. 10.1046/j.0953-816x.2001.01558.x 11359533

[B64] Vela G. Ruiz-gayo M. Fuentes J. A. (1995). Anandamide decreases naloxone-precipitated withdrawal signs in mice chronically treated with morphine. *Neuropharmacology* 34 665–668. 10.1016/0028-3908(95)00032-2 7566503

[B65] Viganò D. Grazia Cascio M. Rubino T. Fezza F. Vaccani A. Di Marzo V. (2003). Chronic morphine modulates the contents of the endocannabinoid, 2-arachidonoyl glycerol, in rat brain. *Neuropsychopharmacology* 28 1160–1167. 12637958 10.1038/sj.npp.1300117

[B66] Viganò D. Rubino T. Vaccani A. Bianchessi S. Marmorato P. Castiglioni C. (2005). Molecular mechanisms involved in the asymmetric interaction between cannabinoid and opioid systems. *Psychopharmacology* 182 527–536. 10.1007/s00213-005-0114-4 16079992

[B67] Wills K. L. Petrie G. N. Millett G. Limebeer C. L. Rock E. M. Niphakis M. J. (2016). Double dissociation of monoacylglycerol lipase inhibition and CB1 antagonism in the central amygdala, basolateral amygdala, and the interoceptive insular cortex on the affective properties of acute naloxone-precipitated morphine withdrawal in rats. *Neuropsychopharmacology* 41 1865–1873. 10.1038/npp.2015.356 26647976 PMC4869055

[B68] Wills K. L. Vemuri K. Kalmar A. Lee A. Limebeer C. L. Makriyannis A. (2014). CB1 antagonism: interference with affective properties of acute naloxone-precipitated morphine withdrawal in rats. *Psychopharmacology* 231 4291–4300. 10.1007/s00213-014-3575-5 24770676 PMC4209202

[B69] Wilson-Poe A. R. Lau B. K. Vaughan C. W. (2014). Repeated morphine treatment alters cannabinoid modulation of GABAergic synaptic transmission within the rat periaqueductal grey. *Br. J. Pharmacol.* 172 681–690. 10.1111/bph.12809 24916363 PMC4292978

[B70] Wilson-Poe A. R. Morgan M. M. Aicher S. A. Hegarty D. M. (2012). Distribution of CB1 cannabinoid receptors and their relationship with mu-opioid receptors in the rat periaqueductal gray. *Neuroscience* 213 191–200. 10.1016/j.neuroscience.2012.03.038 22521830 PMC3367071

[B71] Yamaguchi T. Hagiwara Y. Tanaka H. Sugiura T. Waku K. Shoyama Y. (2001). Endogenous cannabinoid, 2-arachidonoylglycerol, attenuates naloxone-precipitated withdrawal signs in morphine-dependent mice. *Brain Res.* 909 121–126. 10.1016/S0006-8993(01)02655-5 11478928

[B72] Yao L. Mcfarland K. Fan P. Jiang Z. Ueda T. (2006). Adenosine A2a blockade prevents synergy between μ-opiate and cannabinoid CB1 receptors and eliminates heroin-seeking behavior in addicted rats. *Proc. Natl. Acad. Sci. U.S.A.* 103 7877–7882. 10.1073/pnas.0602661103 16684876 PMC1458620

[B73] Young A. M. Katz J. L. Woods J. H. (1981). Behavioral effects of levonantradol and nantradol in the rhesus monkey. *J. Clin. Pharmacol.* 21(8–9 Suppl.), 348S–360S. 10.1002/j.1552-4604.1981.tb02614.x 6271836

[B74] Zaluzny S. G. Chesher G. B. Jackson D. M. Malor R. (1979). The attenuation by Δ9-Tetrahydrocannabinol and morphine of the quasi-morphine withdrawal syndrome in rats. *Psychopharmacology* 61 207–216. 10.1007/BF00426738 86997

